# Supplementary material for: Patterns of Intron Gain and Loss in Fungi
Source: PLoS Biol. 2004 Nov 30;2(12):e422. doi: 10.1371/journal.pbio.0020422 (PMC532390; doi:10.1371/journal.pbio.0020422)
Supplement: Table S1 — Also available at http://genes.mit.edu/NielsenEtAl/. (4.3 MB ZIP). [file pbio.0020422.st001.zip › NielsenEtAl/html/558.html]

AN6175.1.NCU03307.1.MG09889.1.FG00511.1


```
 CLUSTAL W (1.82) Multiple Sequence Alignments - Introns Inserted


Sequence 1: MG09889.1	384 aa
Sequence 2: FG00511.1	350 aa
Sequence 3: NCU03307.1	394 aa
Sequence 4: AN6175.1	336 aa
Alignment Length: 414 aa
Number Identitical Residues: 97 aa
Alignment Score (without introns) 5130


MG09889.1 	---MAHFQLLDPREEE~ELHKSRLLNIEEKPFKRITKRMQTLHAAARWGKQPTPANATTI
NCU03307.1	---MASWGLLDDKEEN~ELHKSRLLNVEEKPFKRVTKRLSTLHS-------LTLSRARQA
FG00511.1 	MGSSASWDLLDPEEEA~ELHKSRLLNVEEKPFKRITKRLNTIS--------------HLL
AN6175.1  	---MTSYGLLDQADED1ALHKTRLLNVEEKPFKRISKRLLNPES--------IIISNATL
          	    : : ***  :*   ***:****:*******::**: .  :                

MG09889.1 	ASPTAADTNQNGTTTSSRPPTSGGAPPPASDELQMLRQELTLDFAAFDSTVARLQFLMRA
NCU03307.1	PTPPPEGNGATAGTTEQEVQTPTVTGDSATSDLAQLKEDIILDFAAFDSSIARLQFLLTA
FG00511.1 	LSPEVVAN----------PPED------LKHKLRELKGEMNLDFDAFNSSITRMQFLHNA
AN6175.1  	PTPPPEGADEDATASAEAEKQK------KLEEWRQFREDASLDFAAFEGSIARIQFLLTS
          	 :*     .  . :: .              .   :: :  *** **:.:::*:***  :

MG09889.1 	NEEERRRYGEDREQILAECEDVRSNTGSLRTQLDEARARLEQRKKFDVLAEGITSNRMLK
NCU03307.1	NERERERYVAERERILNTAQAVRNNTVQLRVQLEQARATLEERKKFDELADKITNNPALR
FG00511.1 	NALERERYKVDQHRILDDCNSVRENNIQLRDQLEAAQATLAQRKKFDELAEKITSNRLLR
AN6175.1  	NEKERERYVAEKERILSTMQAVRNSTVELRVQLEEAQRILALRKSYDEQTEKITSNRLLK
          	*  **.**  ::.:**   : **... .** **: *:  *  **.:*  :: **.*  *:

MG09889.1 	SRADQERNLSKLAEECAQLQEEISQNATTLRERKDQFERIMDEAHRLRRQIRDENDEVDR
NCU03307.1	TRAEQAANLRKLEEEIAELKTESETYGITWHERRDQFAKIMDESMRLRRLIRDEKEEVER
FG00511.1 	PREDQITNLAKLEEECRELERESETYSETWRERREQFNRIMEEGMLLRRQIRDEKEEVDR
AN6175.1  	PREDQQANLQKLQAEIAELEKESKEYAKTWAERREQFGRIVEEGMQLRRLIRDEKEEVER
          	.* :*  ** **  *  :*: * .  . *  **::** :*::*.  *** ****::**:*

MG09889.1 	REGMGD--DEGGEAD-----GHTPRPGIASGNATPLPHAVPQRALAAST-----------
NCU03307.1	REGMGD--DEGGAGDTEADAGQTPRPGISSGNATPRPDSGLPTKSGVESGDAAGTPHPVS
FG00511.1 	REGMNEGADDDAEVDKV---GQTPR-NEPSGNATPHPDGDSQMKTEVDAG----TPNPEA
AN6175.1  	REGMQE--DEDGDDG------ETSK-GKLSGANTPRPDQESSTPSQPGHD----------
          	**** :  *:..  .      .*.: .  **  ** *.           .          

MG09889.1 	-LGSRTPARESPAPQ----DSLKPTPDPVGSFSQGGGRSDSQDGAGATPAASTGAEARIG
NCU03307.1	THGARTPARDTPAPGDENQSFLKPPHDVSGGYSQTTSQEASREGS-ITRGDGNEATQ---
FG00511.1 	LNGGRTPMRDTPGP-----DTLKPQHNRVG--SRGDSQAPSRDASPMPPDDERKAES---
AN6175.1  	-EGDRSPL---PVPT----DKATPGATTPLRQVTTAAEVKQEDATMVDEGEVTGDEDE--
          	  * *:*    * *     .  .*            ..  ..:.:               

MG09889.1 	EGEGEDIEMGDV---GERQPGDDGTPRNTDTQGEGTPRIMVDGPPVVEEKMDTT-
NCU03307.1	---GTDVEMTEVKEDEEEVEEPDSPLSPLPDQTPPRPRITVDGPNADEDTMDTT-
FG00511.1 	---GDDVDMDDS---ESRVHED----------GGATPQVTVEPPEEGEDKMEVDN
AN6175.1  	--RNDDLEEGEE-----------------------------VPDTSNVDKMDTT-
          	   . *::  :                                     :.*:.
```
